# Identification and Antifungal Susceptibility Profiles of *Candida nivariensis* and *Candida bracarensis* in a Multi-Center Chinese Collection of Yeasts

**DOI:** 10.3389/fmicb.2017.00005

**Published:** 2017-01-19

**Authors:** Xin Hou, Meng Xiao, Sharon C.-A. Chen, He Wang, Shu-Ying Yu, Xin Fan, Fanrong Kong, Ying-Chun Xu

**Affiliations:** ^1^Department of Clinical Laboratory, Peking Union Medical College Hospital, Chinese Academy of Medical SciencesBeijing, China; ^2^Graduate School, Peking Union Medical College, Chinese Academy of Medical SciencesBeijing, China; ^3^Centre for Infectious Diseases and Microbiology Laboratory Services, Institute of Clinical Pathology and Medical Research–Pathology West, Westmead Hospital, University of Sydney, SydneyNSW, Australia

**Keywords:** *Candida nivariensis*, *Candida bracarensis*, ITS sequencing, D1/D2 sequencing, ITS sequencer-based capillary gel electrophoresis (SCGE), matrix-assisted laser desorption ionization-time of flight mass spectrometry (MALDI-TOF), antifungal susceptibility

## Abstract

*Candida nivariensis* and *C. bracarensis* are two emerging cryptic species within the *C. glabrata* complex. Thirteen of these isolates from 10 hospitals in China were studied for their species identification and antifungal susceptibilities. Phenotypic and molecular [rDNA ITS sequencing, D1/D2 sequencing and ITS sequencer-based capillary gel electrophoresis (SCGE)] and matrix-assisted laser desorption ionization-time of flight (MALDI-TOF) MS identification methods were compared for their performance in species identification. Twelve of 13 (92.3%) isolates were identified as *C. nivariensis* and one as *C. bracarensis* using ITS sequencing as the reference method. Results obtained by D1/D2 sequencing and ITS SCGE were concordant with ITS sequencing results for all (100%) isolates. SCGE was able to subtype 12 *C. nivariensis* into four ITS SCGE length types. All isolates failed to be identified by the Vitek MALDI-TOF MS system (bioMérieux), whilst the Bruker MS system (Bruker Daltoniks) correctly identified all *C. nivariensis* isolates but using a lowered (≥1.700) cut-off score for species assignment; the *C. bracarensis* isolate was identified but with score <1.700. The Vitek 2 Compact system could not identify 11 *C. nivariensis* and one *C. bracarensis* isolate and misidentified the remaining *C. nivarensis* strain as “*C. glabrata.*” All isolates were susceptible-dose dependent to fluconazole [minimum inhibitory concentration (MIC) range 0.5–4 μg/mL] and were classed as susceptible to echinocandins (MICs ≤ 0.06 μg/mL). All 13 isolates had low MICs for other azoles (MICs ≤ 0.5 μg/mL), amphotericin B (MICs ≤ 2 μg/mL) and 5-flucytosine (MICs ≤ 0.25 μg/mL). Our results reinforce the need for molecular differentiation of species of *C. nivarensis* and *C. bracarensis*. The performance of MALDI-TOF may be improved by adding mass spectral profiles (MSPs) into the current databases. The antifungal susceptibility profile of isolates should be monitored.

## Introduction

*Candida* remains the most important cause of opportunistic mycoses worldwide (Pfaller and Diekema, 2007). Although overall, *C. albicans* remains the most common *Candida* species responsible for invasive candidiasis, non-*albicans Candida* species increasingly represent a significant burden (Pfaller et al., 2010). With evolving and changing fungal taxonomy due to advances in DNA sequencing techniques, previously unrecognized species, as well as “cryptic” species within species complexes have been identified. Examples include the distinction between closely related species within the *C. parapsilosis* species complex (*C. parapsilosis* sensu stricto, *C. orthopsilosis* and *C. metapsilosis*), *C. haemulonii* complex (*C. haemulonii, C. duobushaemulonii* and *C. haemulonii* var. *vulnera*) and *C. glabrata* complex (*C. glabrata* sensu stricto, *C. nivariensis* and *C. bracarensis*; Alcoba-Florez et al., 2005; Tavanti et al., 2005; Correia et al., 2006; Cendejas-Bueno et al., 2012).

Although cryptic species of the *C. glabrata* complex likely cause similar disease manifestations as *C. glabrata* sensu stricto, their distinction is important due to differences in antifungal susceptibility where both *C. nivariensis* and *C. bracarensis* have been reported to be more drug resistant including to the azoles (Fujita et al., 2007; Bishop et al., 2008; Borman et al., 2008; Figueiredo-Carvalho et al., 2016). However, phenotypic identification methods cannot identify these two species where they are “misidentified” as “*C. glabrata”* by identification systems such as Vitek 2 Compact system and API ID32C (bioMérieux, Marcy l’Etoile, France; Alcoba-Florez et al., 2005; Correia et al., 2006; Lockhart et al., 2009). Matrix-assisted laser desorption ionization–time of flight mass spectrometry (MALDI-TOF MS) is able to rapidly and accurately identify *Candida* species (Pinto et al., 2011). The mass spectra (MS) of *C. glabrata* sensu stricto, *C. nivariensis* and *C. bracarensis* are included in most commercial MS databases (Pinto et al., 2011; Gorton et al., 2013). Nonetheless, confirmation of species identification remains reliant on DNA sequencing of the fungal rDNA ITS region (considered the reference method) or the D1/D2 region of the 28S rRNA gene in many instances (Alcoba-Florez et al., 2005; Correia et al., 2006). Other molecular methods may also be employed to identify and distinguish within *C. glabrata* species complex including an ITS-targeted SCGE method. We had previously used SCGE to distinguish between subtypes of both *C. glabrata* (cgl-1 and cgl-2) and *C. nivariensis* (cni-1 and cni-2; Hou et al., 2016).

Despite growing awareness of their clinical significance, data on the occurrence and distribution of *C. nivariensis* and *C. bracarensis* in clinical specimens in China has not been described. Therefore, here we have studied the epidemiology and antifungal susceptibility of *C. nivariensis* and *C. bracarensis* clinical isolates collected from a multi-center surveillance in China over 5 years.

## Materials and Methods

### Ethics Statement

The study was approved by the Human Research Ethics Committee of Peking Union Medical College Hospital (No. S-263). Written informed consent was obtained from patients for the use of the samples in research.

### Yeast Isolates

All isolates were collected as part of the nationwide surveillance program for IFDs in China -CHIF-NET from August 2009 to July 2014 (**Table 1**). The CHIF-NET was a nationwide, prospective, laboratory-based, surveillance network established to provide updated information on the epidemiology of IFDs in China and the study inclusion criteria are previously described (Wang et al., 2012). Isolates were collected from different study centres and then forwarded to a central reference laboratory (Department of Clinical Laboratory, Peking Union Medical College Hospital) for species identification. Species were identified at study sites but confirmed in all cases at the reference laboratory. Specifically, species identification was performed by MALDI-TOF MS and by sequencing of the ITS region according to the algorithm of Zhang et al. (2014). All isolates identified as “*C. glabrata*” and all uncommon or interesting *Candida* species were also identified by sequencing of the ITS regions. Over 8000 *Candida* isolates were collected during 5 years and a total of 12 *C. nivariensis* and one *C. bracarensis* were identified. Then phenotypic and molecular (rDNA ITS sequencing, D1/D2 sequencing and ITS SCGE) and MALDI-TOF MS identification methods were compared and evaluated for their performance in species identification.

**Table 1:** Demographic data and identification of the *Candida nivariensis* and *Candida bracarensis* isolates included in this study.

Strain	Collection date	Gender	Age	Department	Source of isolates	ITS sequencing	MALDI-TOF		D1/D2 sequencing	ITS SCGE	Vitek 2 Compact (Score)
							Vitek MS	Bruker MS (Score)			
10HX033	2010	Male	38	Surgical	Blood	*C. nivariensis*	NI	cni (1.751)	cni	cni-2	Unidentified
10HX034	2010	Female	47	Medical	CSF	*C. nivariensis*	NI	cni (1.728)	cni	cni-2	Unidentified
12HB004	2012	Male	24	Emergency	Blood	*C. nivariensis*	NI	cni (1.777)	cni	cni-2	Unidentified
12TZ215	2012	Male	UN	Surgical	Blood	*C. nivariensis*	NI	cni (1.747)	cni	cni-1	Unidentified
13J3030	2013	Female	82	Surgical	AF	*C. nivariensis*	NI	cni (1.861)	cni	cni-1	Unidentified
13H4102	2013	Female	82	ICU	Blood	*C. nivariensis*	NI	cni (1.784)	cni	cni-2	Unidentified
13H1295	2013	Female	79	ICU	AF	*C. nivariensis*	NI	cni (1.804)	cni	cni-2	Unidentified
13JX046	2013	Male	55	ICU	Blood	*C. nivariensis*	NI	cni (1.916)	cni	cni-3	Unidentified
13AH035	2013	Female	0	Neonatology	Blood	*C. nivariensis*	NI	cni (1.732)	cni	cni-1	*Candida glabrata* (94%)
13AH037	2013	Female	0	Neonatology	Blood	*C. nivariensis*	NI	cni (1.944)	cni	cni-1	Unidentified
13AH038	2013	Male	0	Neonatology	Blood	*C. nivariensis*	NI	cni (1.770)	cni	cni-1	Unidentified
13W3020	2013	Male	73	Urology	Blood	*C. nivariensis*	NI	cni (1.708)	cni	cni-4	Unidentified
12NX009	2012	Female	UN	Surgical	Pus	*C. bracarensis*	NI	cbr (1.447)	cbr	cbr-1	Unidentified

*UN, unkown; CSF, cerebrospinal fluid; AF, ascitic fluid; NI, no identification; ICU, intensive care unit; cni, *C. nivariensis*; cbr, C. bracarensis.*

### Vitek 2 Compact System Identification

All 13 isolates were also re-identified using the Vitek-2 yeast identification card (Vitek-2 Yeast ID; bioMérieux, Marcy l’Etoile, France) following the manufacturer’s instructions. The inoculum suspensions for the Vitek 2 were prepared in sterile saline at turbidity equal to a 2.0 McFarland standard, as measured using a DensiChek instrument (bioMérieux). The individual test cards were automatically filled with the prepared culture suspension, sealed, and incubated by the Vitek 2 instrument. Cards were incubated for 18 h at 35°C and read every 15 min. The final profile results were compared with the database, and the identification of the unknown organism was obtained.

### Sequencer-Based Identification

(i) For DNA sequencing, all isolates were identified by sequencing the ITS region gene and the D1/D2 domain of the 28S rRNA gene was performed as previously described (Amberg et al., 2005; Zhang et al., 2014). The ITS region and D1/D2 sequences of strains used in this study have been deposited in GenBank (Supplementary Table S1).
(ii) For ITS SCGE, both the ITS1 and full-length ITS regions of each isolate were amplified by a duplex PCR as previously described (Hou et al., 2016).

### Phylogenetic Analysis

ITS and D1/D2 nucleotide sequences of *C. nivariensis* and *C. bracarensis* were obtained from GenBank (Supplementary Table S1). *C. glabrata* ATCC 2001 was also included. The ITS and D1/D2 sequences were then used for phylogenetic analysis by the maximum-likelihood algorithm with 1000 bootstrap replication to ensure robustness using MEGA software (version 6.0, MEGA Inc., Englewood, NJ, USA).

### Matrix-Assisted Laser Desorption Ionization-Time of Flight Mass Spectrometry (MALDI-TOF MS) Analysis

All isolates were identified by both Vitek MS system (IVD Knowledgebase version 2.0, bioMérieux, Marcy l’Etoile, France) and Bruker Autoflex Speed TOF/TOF MS system using Biotyper version 3.1 software (Bruker Daltoniks, Billerica, MA, USA) according to the manufacturer’s instructions. For the Vitek MS system, the results were scored in one of three ways, (i) a single identification (confidence value of 60.0 to 99.9%), (ii) a split identification for which a set of possible organisms is displayed, or (iii) NI when no match is found (Zhang et al., 2014). *C. nivariensis* and *C. bracarensis* reference spectra are not included in the Vitek MS v2.0 database. With the Bruker system, identification was provided according to manufacturer-determined criteria: a spectral score of <1.700 was considered not to provide reliable identification. A score of 1.700 but <2.000 indicated identification at the genus level, and a score of ≥2.000, identification at the species level (Deak et al., 2015). The current Bruker Daltonik v3.1 database contains 5,989 MSPs, which include reference spectra of *C. nivariensis* and *C. bracarensis.*

### Antifungal Susceptibility Testing

The *in vitro* susceptibility to nine antifungal drugs – FLC, VRC, ITC, POS, CAS, MCF, ANF, AMB and 5FC – was determined using Sensititre YeastOne^TM^ YO10 methodology (Thermo Scientific, Cleveland, OH, USA) following the manufacturer’s instructions. Briefly, isolates were sub-cultured onto Sabouraud dextrose agar (Oxoid Ltd., Hampshire, UK) and incubated at 35°C for 48 h. After this, 20 μL of 0.5 McFarland yeast suspension was transferred into 11 mL of inoculum broth and then 100 μL of the inoculated broth was transferred to each well of the manufacturer’s plate. Plates were incubated at 35°C and the MIC endpoints were read at 24 h. *C. krusei* ATCC 6258 and *C. parapsilosis* ATCC22019 were used as quality control strains in every test run. For *C. glabrata* complex, MIC values were interpreted according to the CBPs for FLC and echinocandins according to the CLSI M27-S4 standard, and to ECVs for the other agents (Clinical and Laboratory Standards Institute, 2012; Pfaller and Diekema, 2012).

### Review of *C. nivariensis* and *C. bracarensis* Infections Reported in PubMed

For comprehensive understanding of the current epidemiology and antifungal susceptibility profiles of *C. nivariensis* and *C. bracarensis*, we reviewed and summarized all published literature in PubMed ^1^ database as of July 12, 2016.

## Results

Detailed information relating to the study isolates is summarized in **Table 1**. The 13 isolates were collected from 13 patients at 10 hospitals situated in eight provinces across China. For *C. nivariensis*, 25% (3/12) of the isolates were from patients admitted in the surgery department, 25% (3/12) from the ICU, 25% (3/12) from neonatology department, and the remaining 25% (3/12) were from other departments. The *C. bracarensis* isolate was grown from pus from a patient admitted to the surgery department in 2012 (**Table 1**). *C. nivariensis* recovered from blood cultures comprised 75% (9/12) of the strains with two isolates (2/12, 16.7%) recovered from CSF and one (8.3%) from AF.

### Species Identification of *C. nivariensis* and *C. bracarensis* by Sequencer-Based Identification

The ITS sequences of the 13 study isolates exhibited 98.6 to 99.7% and 98.5% sequence identity to ITS sequences of reference isolates archived in the GenBank database (*C. nivariensis* CBS 10161 and *C. bracarensis* 153M^T^; Supplementary Table S1). Twelve isolates were identified as *C. nivariensis* and one as *C. bracarensis* (**Table 1**).

Analysis of the D1/D2 gene region sequences identified all 13 clinical isolates with 100% concordance with ITS sequencing results (**Table 1**). The D1/D2 region sequence of the 12 *C. nivariensis* isolate (KX499363 to KX499375) showed 99.6% sequence identity to those of *C. nivariensis* isolates in GenBank (*C. nivariensis* CBS 10161, accession no. EF056323.1) and the *C. bracarensis* sequence showed 99.7% sequence identity to that of *C. bracarensis* 153M^T^ (accession no. AY589572.1). Results of ITS SCGE identified lengths of ITS1 and full-length ITS regions for *C. nivariensis* and *C. bracarensis*, which were identified as “cni-1,” “cni-2,” “cni-3,” “cni-4” and “cbr-1” as previously described (Hou et al., 2016).

### Phylogenetic Analysis

**FIGURE 1:**
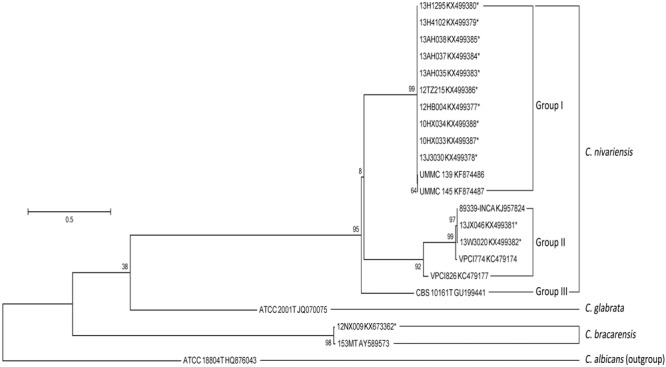
**The maximum-likelihood tree of *C. nivariensis* and *C. bracarensis* with ITS sequences available in GenBank, using *Candida albicans* ATCC 18804 as an outgroup**.

**FIGURE 2:**
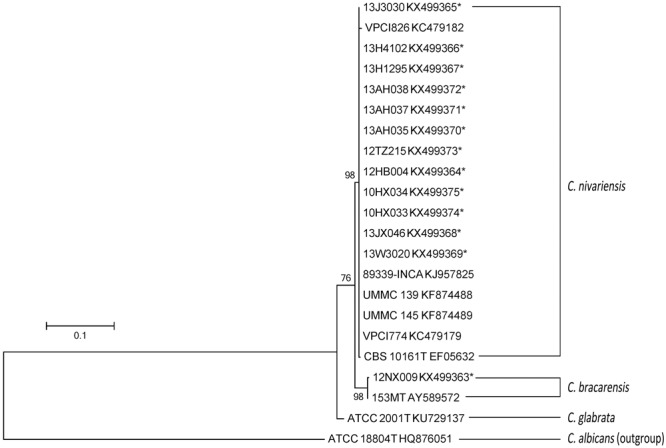
**The maximum-likelihood tree of *C. nivariensis* and *C. bracarensis* with D1/D2 sequences available in GenBank, using *Candida albicans* ATCC 18804 as an outgroup**.

The nucleotide sequence alignments within *C. nivariensis* and *C. bracarensis*, with reference to sequences of *C. nivariensis* type strain CBS 10161 and *C. bracarensis* type strain 153M^T^ as the reference points, showed these species had high inter-species genetic similarity within ITS region (98.4 to 100%) and D1/D2 region (98.1 to 99.8%; Supplementary Table S1). The maximum-likelihood analysis of the ITS and D1/D2 region yielded similar results (**Figures 1** and **2**). For ITS, three groups of *C. nivariensis* were distinguished and our 12 isolates were in Groups I and II (**Figure 1**). Intra-species ITS diversity in *Candida* species has been described before (Merseguel et al., 2015). There was no intra-species D1/D2 diversity in 12 *C. nivariensis* isolates (**Figure 2**). Both ITS and D1/D2 sequencing can be used for the differentiation of *C. glabrata, C. nivariensis* and *C. bracarensis*.

### Performance of Vitek-2 Compact and Two MALDI-TOF MS Systems

As compared with ITS sequencing, 11 *C. nivariensis* and the *C. bracarensis* isolates could not be identified by the Vitek 2 Compact system (bioMérieux). The remaining *C. nivariensis* was misidentified as *C. glabrata* (score = 94%; **Table 1**). All isolates were not identified by the Vitek MS system because *C. nivariensis* and *C. bracarensis* reference spectra were not included in the Vitek MS v2.0 database (**Table 1**). However, the Bruker system correctly identified all *C. nivariensis* isolates to the genus level with a MS score of <2.000 but ≥1.700. The *C. bracarensis* isolate was identified as such but with a MS score of 1.447 (**Table 1**).

### Antifungal Susceptibility

The antifungal susceptibilities of the study isolates to nine antifungal agents are shown in **Table 2**. All isolates were S-DD to FLC (MIC range 0.5–4 μg/mL) and were classed as susceptible to echinocandins (MICs ≤ 0.06 μg/mL). All 13 isolates were also wild type to VRC (MICs ≤ 0.12 μg/mL), ITC (MICs ≤ 0.25 μg/mL), POS (MICs ≤ 0.5 μg/mL), AMB (MICs ≤ 2 μg/mL) and 5FC (MICs ≤ 0.25 μg/mL).

**Table 2:** *In vitro* susceptibility of 12 *Candida nivariensis* and one *Candida bracarensis* to nine antifungal agents.

Strain or parameter	MIC (μg/mL)								
	ANF	MCF	CAS	5FC	POS	VRC	ITC	FLC	AMB
***C. nivariensis***									
10HX033	0.03	≤0.008	0.03	0.25	0.25	0.03	0.12	1	2
10HX034	0.06	0.015	0.03	0.12	0.25	0.03	0.12	1	1
12HB004	0.03	0.015	0.03	0.12	0.12	0.015	0.12	0.5	1
12TZ215	0.06	0.015	0.06	0.25	0.25	0.03	0.12	0.5	1
13J3030	0.015	≤0.008	0.06	0.12	0.12	0.03	0.06	1	0.5
13H4102	0.03	0.03	0.06	0.12	0.5	0.03	0.12	0.5	1
13H1295	0.03	0.015	0.03	0.25	0.25	0.06	0.25	2	2
13JX046	0.06	0.015	0.06	0.25	0.12	0.03	0.12	1	1
13AH035	0.06	0.015	0.06	0.12	0.25	0.03	0.12	1	1
13AH037	0.06	0.015	0.06	0.12	0.25	0.015	0.12	1	1
13AH038	0.06	0.015	0.06	0.25	0.25	0.03	0.12	1	2
13W3020	0.06	0.015	0.06	0.25	0.5	0.12	0.25	4	1
MIC range	0.015-0.06	≤0.008-0.03	0.03-0.06	0.12-0.25	0.12-0.5	0.015-0.12	0.06-0.25	0.5-4	0.5-2
MIC_50_	0.06	0.015	0.06	0.12	0.25	0.03	0.12	1	1
MIC_90_	0.06	0.015	0.06	0.25	0.5	0.06	0.25	2	2
GM	0.04	0.01	0.05	0.17	0.23	0.03	0.13	1	1.12
***C. bracarensis***									
12NX009	0.03	0.015	0.03	0.25	0.25	0.03	0.12	1	2

*FLC, fluconazole; VRC, voriconazole; ITC, itraconazole; POS, posaconazole; CAS, caspofungin; MCF, micafungin; ANF, anidulafungin; AMB, amphotericin B; 5FC, 5-flucytosine.*

### *C. nivariensis* and *C. bracarensis* Reported in PubMed

By searching the literature in PubMed^1^ using “*C. nivariensis* or *C. bracarensis*” as the keywords, a total of 46 articles were found as of July 12, 2016. Among these, 15 and seven, full text articles reporting on the detection of *C. nivariensis* and *C. bracarensis* isolates were found, respectively; with 65 cases identified in 12 regions and 16 cases identified in seven regions, respectively (Supplementary Table S1). *C. nivariensis* caused mucosal superficial disease as well as IFDs and have been isolated from body sites including the mouth, toenails, urine, sputum, vagina, CSF, blood, BAL and other sterile body fluids. Two *C. nivariensis* isolates have been recovered from soil and bark. *C. bracarensis* has been isolated from throat, stool, abscess, sputum, vagina, blood and other sterile body fluids.

Data on *C. nivariensis* (12 articles were found) and *C. bracarensis* (six articles) antifungal susceptibility profiles are relatively limited. An overview of the published data is presented in Supplementary Table S2. Some strains exhibited high MICs to FLC alone or to all azoles, suggesting that the same mechanisms of resistance as found in *C. glabrata* may be involved (Fujita et al., 2007; Bishop et al., 2008; Borman et al., 2008; Gorton et al., 2013; Figueiredo-Carvalho et al., 2016). Further studies, using the *C. nivariensis* and *C. bracarensis* genome data, that are now available, might be useful to confirm this hypothesis (Angoulvant et al., 2016). MICs to echinocandins of *C. nivariensis* and *C. bracarensis* are overall low (MIC_90_ < 0.5 μg/ml for CAS; Supplementary Table S2). To date there are no reports on emergence of echinocandin-resistant *C. nivariensis* or *C. bracarensis* strains.

## Discussion

*Candida nivariensis* and *C. bracarensis* are reportedly uncommon amongst clinical *Candida* isolates. Although the three species are closely related phylogenetically, DNA sequencing has shown that *C. glabrata, C. nivariensis* and *C. bracarensis* are sufficiently genetically distinct to justify their assignment as separate species (**Figures 1** and **2**) (Alcoba-Florez et al., 2005; Correia et al., 2006; Mirhendi et al., 2011).

In the present study, we have estimated the proportion of *C. nivariensis* and *C. bracarensis* amongst *Candida* strains cultured from patient with IFDs in China. Overall, during the 5-year CHIF-NET study period, *C. nivariensis* and *C. bracarensis* collectively represented only 0.11% (13/9673) of all yeast isolates, while 947 (9.79%, 947/9673) *C. glabrata* were collected. Among the 13 isolates, *C. nivariensis* (12/9673) was more frequently isolated compared with *C. bracarensis* (1/9673), which is in accordance with a retrospective study from four French university hospitals (2010–2014) where *C. nivariensis* and *C. bracarensis* were, respectively, identified in 0.12 and 0.01% of the 55,464 yeast strains (Angoulvant et al., 2016). Conversely, in the ARTEMIS DISK Global Antifungal Surveillance Study (2001–2006), where a total of 1,598 isolates phenotypically identified as *C. glabrata* were re-tested by peptide nucleic acid fluorescence *in situ* hybridization, only one *C. nivariensis* and two *C. bracarensis* were identified (Lockhart et al., 2009). In a Spanish study over a period of 2 years (2008–2009), a total of three (2%) of 143 *C. glabrata* clinical strains were identified as *C. bracarensis* and none as *C. nivariensis* (Cuenca-Estrella et al., 2011). Data for prevalence of *C. nivariensis* and *C. bracarensis* are directly influenced by the identification method used and comparisons between studies need to take into account the different identification methodologies used. In this study we have reinforced that molecular methods are the preferred approach for definitive identification of members within the species complex. Currently, ITS and D1/D2 regions were considered as universal DNA barcode markers for *Candida* spp. identification (Taverna et al., 2013). In this study, ITS sequencing was able to identify all 13 isolates and of note, concordance of results with ITS sequencing was 100% with those obtained by D1/D2 sequencing and ITS SCGE. An ITS SCGE assay has also shown promise for identification of these cryptic species and indeed to identify subtypes within these species. Twelve *C. nivariensis* were subtyped into four different ITS LTs, i.e., five cni-1, five cni-2, one cni-3 and one cni-4. The technique has the advantage of being simple to use (Hou et al., 2016).

Conventional phenotypic-based methods of classification of species within the *C. glabrata* complex have proven ineffective in accurately identifying these species as was the case in the present study (Alcoba-Florez et al., 2005; Correia et al., 2006), where all 13 isolates could not be identified by the Vitek 2 system (bioMérieux), except for one isolate “misidentified” as *C. glabrata.* Hence any unusual identification result should be confirmed by an alternative more discriminatory method. One simple method is to visualize the color of colonies on CHROMagar (Becton Dickinson, Heidelberg, Germany). *C. nivariensis* and *C. bracarensis* isolates yield white colonies on CHROMagar in contrast to the purple colonies of *C. glabrata* (Lockhart et al., 2009), and may be useful as a screen for members of the *C. glabrata* clade.

MALDI-TOF MS has become a routine identification tool for the identification of yeast and yeast-like organisms in many laboratories (Pinto et al., 2011; Gorton et al., 2013). In one study, four isolates collected from vagina were identified as *C. nivariensis* by the Bruker MS system (Bruker Daltoniks), with a log score between 1.802 and 2.086 (Aznar-Marin et al., 2015). MALDI-TOF MS has been reported to have the ability to distinguish within the *C. glabrata* clade, i.e., between *C. glabrata, C. nivariensis* and *C. bracarensis*, with scores of <1.700 as *C. nivariensis* (3/10 strains) and *C. bracarensis* (1/1 strain; Pinto et al., 2011). We found the database of the Vitek MS system (bioMérieux; IVD Knowledgebase version 2.0) to be limited by the absence of reference spectra for *C. nivariensis* and *C. bracarensis*. In comparison, the Bruker system (Bruker Daltoniks) was able to distinguish between all three members of the *C. glabrata* complex; the log scores of all *C. nivariensis* isolates were >1.700 but <2.000, while the log score of the *C. bracarensis* isolate was <1.700. The current Bruker Daltonik v3.1 database contains six MSPs for *C. nivariensis* and two MSPs for *C. bracarensis.* The addition of further reference spectra to the database may improve the utility of MALDI-TOF MS-based identification (Pinto et al., 2011). To improve the identification capacity of MALDI-TOF MS, it is important for MS databases to contain more reference mass spectra from type strains of different species, and also spectra representing different strains of the same species (Singhal et al., 2015).

Accurate identification of *C. nivariensis* and *C. bracarensis* in clinical samples is important, because the antifungal susceptibility patterns of *C. nivariensis* and *C. bracarensis* may differ from that of *C. glabrata*. *In vitro* susceptibility to azoles and 5FC appears to be lower for *C. nivariensis* than for *C. glabrata* (Fujita et al., 2007; Bishop et al., 2008; Borman et al., 2008). Although our isolates had low MICs to the azoles, echinocandins, AMB and 5FC, several studies have reported azoles resistance in these two species (Fujita et al., 2007; Bishop et al., 2008; Borman et al., 2008; Gorton et al., 2013; Figueiredo-Carvalho et al., 2016). For *C. nivariensis*, the MICs range of FLC has been 0.5 – >256 μg/ml, ITC MICs range 0.03 – >16 μg/ml, while the MICs range of FLC of *C. bracarensis* was 2–256 μg/ml and for ITC, 0.06 – >16 μg/ml (Supplementary Table S2). All isolates reported thus far have been susceptible to the echinocandins. Additional strains of *C. nivariensis* and *C. bracarensis* need to be studied to better understand their susceptibility profile and to determine whether these species have clinically significant differences in responses to antifungal therapy compared to *C. glabrata.* Moreover, studies from multiple geographical locations are needed to elucidate the epidemiology of infection, colonization and antifungal resistance of *C. nivariensis* and *C. bracarensis.*

## Summary

This is the first systemic study regarding the epidemiology, identification and antifungal susceptibility profiles of *C. nivariensis* and *C. bracarensis* isolates in China. Our study reinforces the need for molecular identification of these two new and rarely species. Further improvements in the MALDI-TOF databases are needed to increase the identification accuracy. Studies from multiple geographical locations and additional data are required to better characterize their frequency, geographical distribution, susceptibility profiles and clinical features of infections due to *C. nivariensis* and *C. bracarensis*.

## Author Contributions

XH, MX, and Y-CX conceived and designed the experiments, performed the experiments, analyzed the data, and wrote the paper. HW, S-YY, and XF performed the experiments and analyzed the data. SC and FK revised the paper critically for important intellectual content. XH, MX, SC, HW, S-YY, XF, FK, and Y-CX read and approved the final version of the manuscript.

## Conflict of Interest Statement

The authors declare that the research was conducted in the absence of any commercial or financial relationships that could be construed as a potential conflict of interest.

## Footnotes

1 http://www.ncbi.nlm.nih.gov/pubmed

## Abbreviations

5FC: 5-flucytosine
AF: ascitic fluid
AMB: amphotericin B
ANF: anidulafungin
BAL: bronchoalveolar lavage fluid
CAS: caspofungin
CBPs: clinical breakpoints
cbr: *C. bracarensis*
CHIF-NET: China Hospital Invasive Fungal Surveillance Net
CLSI: Clinical and Laboratory Standards Institute
cni: *C. nivariensis*
CSF: cerebrospinal fluid
ECVs: epidemiological cut-off values
FLC: fluconazole
ICU: intensive care unit
IFDs: invasive fungal diseases
ITC: itraconazole
ITS LTs: ITS length types
ITS: internal transcribed spacer
MALDI-TOF: matrix-assisted laser desorption ionization-time of flight
MCF: micafungin
MIC: minimum inhibitory concentration
MSPs: mass spectral profiles
NI: no identification
POS: posaconazole
SCGE: sequencer-based capillary gel electrophoresis
S-DD: susceptible-dose dependent
STs: sequence types
UN: unkown
VRC: voriconazole
