# Supplementary material for: Identification and Antifungal Susceptibility Profiles of Candida nivariensis and Candida bracarensis in a Multi-Center Chinese Collection of Yeasts
Source: Front Microbiol. 2017 Jan 19;8:5. doi: 10.3389/fmicb.2017.00005 (PMC5243801; doi:10.3389/fmicb.2017.00005)
Supplement: Supplementary file 1 [file Table_1.DOCX]

**Table S1 Summary of *Candida nivariensis* and *Candida bracarensis* isolates from this study, published literatures or GenBank, and genetic comparison within these two species**

| **Strain** | **Region** | **Collection date** | **Origin** | **ITS GenBank accession no. (Length of ITS in bp)** | **Identity (%)** | **D1/D2 GenBank accession no. (Length of D1/D2 in bp)** | **Identity (%)** | **Reference** |
| --- | --- | --- | --- | --- | --- | --- | --- | --- |
| ***C. nivariensis* type strain** | |  |  |  |  |  |  |  |
| CBS 10161 | Indonesia | 2004 | Oral rinse | GU199441.1 (757) | Reference sequence | EF056323.1 (550) | Reference sequence | ([Wahyuningsih et al., 2008](#_ENREF_18)) |
| ***C. bracarensis* type strain** | |  |  |  |  |  |  |  |
| 153M^T^ | Portugal | 2004 | Vaginal exudate | AY589573.2 (746) | Reference sequence | AY589572.1 (581) | Reference sequence | ([Correia et al., 2006](#_ENREF_5)) |
| ***C. nivariensis* clinical strain** | | |  |  |  |  |  |  |
| 10HX033 | China | 2010 | Blood | KX499387 (736) | 728/736 (98.9) | KX499374 (603) | 548/550 (99.6) | This study |
| 10HX034 | China | 2010 | CSF | KX499388 (736) | 728/736 (98.9) | KX499375 (603) | 548/550 (99.6) | This study |
| 12HB004 | China | 2012 | Blood | KX499377 (736) | 728/736 (98.9) | KX499364 (603) | 548/550 (99.6) | This study |
| 12TZ215 | China | 2012 | Blood | KX499386 (735) | 728/735 (99.0) | KX499373 (603) | 548/550 (99.6) | This study |
| 13J3030 | China | 2013 | AF | KX499378 (735) | 728/735 (99.0) | KX499365 (603) | 548/550 (99.6) | This study |
| 13H4102 | China | 2013 | Blood | KX499379 (736) | 728/736 (98.9) | KX499366 (603) | 548/550 (99.6) | This study |
| 13H1295 | China | 2013 | AF | KX499380 (736) | 728/736 (98.9) | KX499367 (603) | 548/550 (99.6) | This study |
| 13JX046 | China | 2013 | Blood | KX499381 (736) | 734/736 (99.7) | KX499368 (603) | 548/550 (99.6) | This study |
| 13AH035 | China | 2013 | Blood | KX499383 (735) | 725/735 (98.6) | KX499370 (603) | 548/550 (99.6) | This study |
| 13AH037 | China | 2013 | Blood | KX499384 (735) | 725/735 (98.6) | KX499371 (603) | 548/550 (99.6) | This study |
| 13AH038 | China | 2013 | Blood | KX499385 (735) | 725/735 (98.6) | KX499372 (603) | 548/550 (99.6) | This study |
| 13W3020 | China | 2013 | Blood | KX499382 (737) | 733/737 (99.5) | KX499369 (603) | 548/550 (99.6) | This study |
| HC5937-63 | Spain | 1999 | BAL | AY620959 (529) | 522/526 (99.2) | AY627307 (547) | 545/549 (99.3) | ([Alcoba-Florez et al., 2005](#_ENREF_1)) |
| HC4292-20 | Spain | 2001 | Blood | AY620957 (529) | 522/526 (99.2) | AY627305 (547) | 545/549 (99.3) | ([Alcoba-Florez et al., 2005](#_ENREF_1)) |
| HC7609-30 | Spain | 2002 | Urine | AY620958 (529) | 522/526 (99.2) | AY627306 (547) | 545/549 (99.3) | ([Alcoba-Florez et al., 2005](#_ENREF_1)) |
| K16661 | Japan | 2006 | Blood | NS | NS | NS | NS | ([Fujita et al., 2007](#_ENREF_9)) |
| 8842 | UK | 2006 | Mouth | NS | NS | AM745268 (365) | 319/320 (99.7) | ([Borman et al., 2008](#_ENREF_3)) |
| 8843 | UK | 2006 | Pelvic collection | NS | NS | AM745270 (365) | 319/320 (99.7) | ([Borman et al., 2008](#_ENREF_3)) |
| 8844 | UK | 2005 | Blood | NS | NS | AM745272 (365) | 319/320 (99.7) | ([Borman et al., 2008](#_ENREF_3)) |
| 8845 | UK | 2005 | Mouth | NS | NS | AM745274 (365) | 319/320 (99.7) | ([Borman et al., 2008](#_ENREF_3)) |
| *C. nivariensis* 5 | UK | 2005 | NS | NS | NS | NS | NS | ([Borman et al., 2008](#_ENREF_3)) |
| 8846 | UK | 2005 | NS | NS | NS | AM745276 (365) | 319/320 (99.7) | ([Borman et al., 2008](#_ENREF_3)) |
| *C. nivariensis* 7 | UK | 2005 | Pelvic abscess | NS | NS | NS | NS | ([Borman et al., 2008](#_ENREF_3)) |
| 8847 | UK | 2005 | AF | NS | NS | AM745278 (365) | 319/320 (99.7) | ([Borman et al., 2008](#_ENREF_3)) |
| *C. nivariensis* 9 | UK | 2005 | NS | NS | NS | AM745280 (365) | 319/320 (99.7) | ([Borman et al., 2008](#_ENREF_3)) |
| 8848 | UK | 2006 | Mouth | NS | NS | AM745282 (365) | 319/320 (99.7) | ([Borman et al., 2008](#_ENREF_3)) |
| 8849 | UK | 2005 | Exit site swab | NS | NS | AM745284 (365) | 319/320 (99.7) | ([Borman et al., 2008](#_ENREF_3)) |
| 8850 | UK | 2006 | Peritoneal fluid | NS | NS | AM745286 (365) | 319/320 (99.7) | ([Borman et al., 2008](#_ENREF_3)) |
| 8851 | UK | 2006 | Lung biopsy | NS | NS | AM745288 (365) | 319/320 (99.7) | ([Borman et al., 2008](#_ENREF_3)) |
| 8852 | UK | 2006 | Blood | NS | NS | AM745290 (365) | 319/320 (99.7) | ([Borman et al., 2008](#_ENREF_3)) |
| 8853 | UK | 2006 | NS | NS | NS | AM745292 (364) | 318/320 (99.4) | ([Borman et al., 2008](#_ENREF_3)) |
| *C. nivariensis* 16 | UK | 2006 | Blood | NS | NS | NS | NS | ([Borman et al., 2008](#_ENREF_3)) |
| 2001-2006 | Australia | NS | Pleural fluid | NS | NS | NS | NS | ([Lockhart et al., 2009](#_ENREF_12)) |
| VPCI 32/08 | India | 2008 | Sputum | FM955316 (765) | 755/760 (99.3) | FM955317 (625) | 548/551 (99.5) | ([Chowdhary et al., 2010](#_ENREF_4)) |
| VPCI 1293/08 | India | 2008 | Blood | NS | NS | NS | NS | ([Chowdhary et al., 2010](#_ENREF_4)) |
| URCn1 | UK | 2008 | Urine | NS | | NS | NS | ([Gorton et al., 2013](#_ENREF_10)) |
| URCn2 | UK | 2009 | Urine | NS | NS | NS | NS | ([Gorton et al., 2013](#_ENREF_10)) |
| URCn3 | UK | 2009 | Urine | NS | NS | NS | NS | ([Gorton et al., 2013](#_ENREF_10)) |
| URCn4 | UK | 2009 | Urine | NS | NS | NS | NS | ([Gorton et al., 2013](#_ENREF_10)) |
| URCn5 | UK | 2010 | Urine | NS | NS | NS | NS | ([Gorton et al., 2013](#_ENREF_10)) |
| NS | Spain | 2012 | Blood | NS | NS | NS | NS | ([Lopez-Soria et al., 2013](#_ENREF_13)) |
| VPCI774/P/12 | India | 2011-2012 | Vagina | KC479174 (695) | 686/695 (98.7) | KC479179 (407) | 377/378 (99.7) | ([Sharma et al., 2013](#_ENREF_15)) |
| VPCI785/P/12 | India | 2011-2012 | Vagina | KC479175 (626) | 619/626 (98.9) | KC479180 (453) | 414/422 (98.1) | ([Sharma et al., 2013](#_ENREF_15)) |
| VPCI818/P/12 | India | 2011-2012 | Vagina | KC479176 (608) | 601/608 (98.8) | KC479181 (459) | 414/420 (98.6) | ([Sharma et al., 2013](#_ENREF_15)) |
| VPCI826/P/12 | India | 2011-2012 | Vagina | KC479177 (693) | 685/696 (98.4) | KC479182 (554) | 520/528 (98.5) | ([Sharma et al., 2013](#_ENREF_15)) |
| VPCI1028/12 | India | 2011-2012 | BAL | KC479178 (691) | 684/691 (99.0) | NS | NS | ([Sharma et al., 2013](#_ENREF_15)) |
| 7* | China | 2003-2012 | Vagina | NS | NS | NS | NS | ([Li et al., 2014](#_ENREF_11)) |
| 8* | Poland | 2010-2012 | Urine | NS | NS | NS | NS | ([Swoboda-Kopec et al., 2014](#_ENREF_16)) |
| 3* | Poland | 2010-2012 | Lower respiratory tract | NS | NS | NS | NS | ([Swoboda-Kopec et al., 2014](#_ENREF_16)) |
| 1* | Poland | 2010-2012 | Surgical specimens | NS | NS | NS | NS | ([Swoboda-Kopec et al., 2014](#_ENREF_16)) |
| 1* | Poland | 2010-2012 | Body fluids | NS | NS | NS | NS | ([Swoboda-Kopec et al., 2014](#_ENREF_16)) |
| UMMC 139 | Malaysia | 2013 | Blood | KF874486 (656) | 648/656 (98.8) | KF874488 (519) | 518/520 (99.6) | ([Tay et al., 2014](#_ENREF_17)) |
| UMMC 145 | Malaysia | 2013 | Vagina | KF874487 (656) | 648/656 (98.8) | KF874489 (519) | 518/520 (99.6) | ([Tay et al., 2014](#_ENREF_17)) |
| NS | China | 2012-2013 | Toenails | NS | NS | NS | NS | ([Feng et al., 2015](#_ENREF_7)) |
| 89339/INCA | Brazil | 2004 | Nasal secretion | KJ957824 (731) | 728/731 (99.6) | KJ957825 (603) | 548/550 (99.6) | ([Figueiredo-Carvalho et al., 2016](#_ENREF_8)) |
| SSK1 | Thailand | 2007 | Soil from forest | NS | NS | AB499984 (581) | 548/550 (99.6) | ([Mirhendi et al., 2011](#_ENREF_14)) |
| UB4 | Thailand | 2007 | Bark from forest | NS | NS | AB500875 (581) | 548/550 (99.6) | ([Mirhendi et al., 2011](#_ENREF_14)) |
| CBS 9983 | Iran | 2009 | NS | GU199443 (760) | 757/760 (99.6) | NS | NS | ([Mirhendi et al., 2011](#_ENREF_14)) |
| H41019 | Iran | 2009 | NS | GU199442 (759) | 751/759 (98.9) | NS | NS | ([Mirhendi et al., 2011](#_ENREF_14)) |
| CBS 9985 | Iran | 2009 | NS | GU199444 (760) | 757/760 (99.6) | NS | NS | ([Mirhendi et al., 2011](#_ENREF_14)) |
| CBS 9984 | Iran | 2009 | NS | GU199445 (760) | 757/760 (99.6) | NS | NS | ([Mirhendi et al., 2011](#_ENREF_14)) |
| ***C. bracarensis* clinical strain** | |  |  |  |  |  |  |  |
| 12NX009 | China | 2012 | Pus | KX673362 (741) | 731/742 (98.5) | KX499363 (595) | 579/581 (99.7) | This study |
| NCYC 3133 | UK | 2003 | Blood | NS | NS | AJ582736 (558) | 557/558 (99.8) | ([Correia et al., 2006](#_ENREF_5)) |
| Cagl-78 | USA | 2006 | Stool | NS | NS | NS | NS | ([Bishop et al., 2008](#_ENREF_2)) |
| Cagl-112 | USA | 2006 | Abscess | NS | NS | NS | NS | ([Bishop et al., 2008](#_ENREF_2)) |
| Cagl-121 | USA | 2006 | Throat | NS | NS | NS | NS | ([Bishop et al., 2008](#_ENREF_2)) |
| NRRLY-27794 | USA | 2006 | NS | NS | NS | NS | NS | ([Bishop et al., 2008](#_ENREF_2)) |
| NS | USA | 2002 | Sputum | NS | NS | NS | NS | ([Lockhart et al., 2009](#_ENREF_12)) |
| NS | USA | 2004 | Blood | NS | NS | NS | NS | ([Lockhart et al., 2009](#_ENREF_12)) |
| Clinical isolate 1 | Canada | 2010 | Blood | NS | NS | NS | NS | ([Warren et al., 2010](#_ENREF_19)) |
| Clinical isolate 2 | Canada | 2010 | Blood | NS | NS | NS | NS | ([Warren et al., 2010](#_ENREF_19)) |
| CNM-CL-7030 | Spain | 2008 | Catheter exudate | NS | NS | NS | NS | ([Cuenca-Estrella et al., 2011](#_ENREF_6)) |
| CNM-CL-7326 | Spain | 2009 | Pleural fluid | NS | NS | NS | NS | ([Cuenca-Estrella et al., 2011](#_ENREF_6)) |
| CNM-CL-7380 | Spain | 2009 | Blood | NS | NS | NS | NS | ([Cuenca-Estrella et al., 2011](#_ENREF_6)) |
| NS | China | 2003-2012 | Vagina | NS | NS | NS | NS | ([Li et al., 2014](#_ENREF_11)) |
| advanDx1 | Iran | 2009 | NS | GU199438 (805) | 735/747 (98.4) | NS | NS | ([Mirhendi et al., 2011](#_ENREF_14)) |
| advanDx2 | Iran | 2009 | NS | GU199439 (805) | 746/746 (100) | NS | NS | ([Mirhendi et al., 2011](#_ENREF_14)) |

* Number of isolates

Abbreviations: CSF: cerebrospinal fluid; AF: ascitic fluid; BAL: Bronchoalveolar lavage fluid; NS, not stated

**Reference**

Alcoba-Florez, J., Mendez-Alvarez, S., Cano, J., Guarro, J., Perez-Roth, E., and del Pilar Arevalo, M. (2005). Phenotypic and molecular characterization of *Candida nivariensis* sp. nov., a possible new opportunistic fungus. *J Clin Microbiol* 43(8)**,** 4107-4111. doi: 10.1128/JCM.43.8.4107-4111.2005.

Bishop, J.A., Chase, N., Magill, S.S., Kurtzman, C.P., Fiandaca, M.J., and Merz, W.G. (2008). *Candida bracarensis* detected among isolates of *Candida glabrata* by peptide nucleic acid fluorescence in situ hybridization: susceptibility data and documentation of presumed infection. *J Clin Microbiol* 46(2)**,** 443-446. doi: 10.1128/JCM.01986-07.

Borman, A.M., Petch, R., Linton, C.J., Palmer, M.D., Bridge, P.D., and Johnson, E.M. (2008). *Candida nivariensis*, an emerging pathogenic fungus with multidrug resistance to antifungal agents. *J Clin Microbiol* 46(3)**,** 933-938. doi: 10.1128/JCM.02116-07.

Chowdhary, A., Randhawa, H.S., Khan, Z.U., Ahmad, S., Juneja, S., Sharma, B., et al. (2010). First isolations in India of *Candida nivariensis*, a globally emerging opportunistic pathogen. *Medical Mycology* 48(2)**,** 416-420. doi: 10.3109/13693780903114231.

Correia, A., Sampaio, P., James, S., and Pais, C. (2006). *Candida bracarensis* sp. nov., a novel anamorphic yeast species phenotypically similar to *Candida glabrata*. *Int J Syst Evol Microbiol* 56(Pt 1)**,** 313-317. doi: 10.1099/ijs.0.64076-0.

Cuenca-Estrella, M., Gomez-Lopez, A., Isla, G., Rodriguez, D., Almirante, B., Pahissa, A., et al. (2011). Prevalence of *Candida bracarensis* and *Candida nivariensis* in a Spanish collection of yeasts: comparison of results from a reference centre and from a population-based surveillance study of candidemia. *Med Mycol* 49(5)**,** 525-529. doi: 10.3109/13693786.2010.546373.

Feng, X., Ling, B., Yang, X., Liao, W., Pan, W., and Yao, Z. (2015). Molecular identification of *Candida* species isolated from onychomycosis in Shanghai, China. *Mycopathologia* 180(5-6)**,** 365-371. doi: 10.1007/s11046-015-9927-9.

Figueiredo-Carvalho, M.H., Ramos Lde, S., Barbedo, L.S., Chaves, A.L., Muramoto, I.A., Santos, A.L., et al. (2016). First description of *Candida nivariensis* in Brazil: antifungal susceptibility profile and potential virulence attributes. *Mem Inst Oswaldo Cruz* 111(1)**,** 51-58. doi: 10.1590/0074-02760150376.

Fujita, S., Senda, Y., Okusi, T., Ota, Y., Takada, H., Yamada, K., et al. (2007). Catheter-related fungemia due to fluconazole-resistant *Candida nivariensis*. *J Clin Microbiol* 45(10)**,** 3459-3461. doi: 10.1128/JCM.00727-07.

Gorton, R.L., Jones, G.L., Kibbler, C.C., and Collier, S. (2013). *Candida nivariensis* isolated from a renal transplant patient with persistent candiduria-molecular identification using ITS PCR and MALDI-TOF. *Med Mycol Case Rep* 2**,** 156-158. doi: 10.1016/j.mmcr.2013.10.001.

Li, J., Shan, Y., Fan, S., and Liu, X. (2014). Prevalence of *Candida nivariensis* and *Candida bracarensis* in vulvovaginal Candidiasis. *Mycopathologia* 178(3-4)**,** 279-283. doi: 10.1007/s11046-014-9800-2.

Lockhart, S.R., Messer, S.A., Gherna, M., Bishop, J.A., Merz, W.G., Pfaller, M.A., et al. (2009). Identification of *Candida nivariensis* and *Candida bracarensis* in a large global collection of *Candida glabrata* isolates: comparison to the literature. *J Clin Microbiol* 47(4)**,** 1216-1217. doi: 10.1128/JCM.02315-08.

Lopez-Soria, L.M., Bereciartua, E., Santamaria, M., Soria, L.M., Hernandez-Almaraz, J.L., Mularoni, A., et al. (2013). First case report of catheter-related fungemia by *Candida nivariensis* in the Iberian Peninsula. *Rev Iberoam Micol* 30(1)**,** 69-71. doi: 10.1016/j.riam.2012.09.001.

Mirhendi, H., Bruun, B., Schonheyder, H.C., Christensen, J.J., Fuursted, K., Gahrn-Hansen, B., et al. (2011). Differentiation of *Candida glabrata, C. nivariensis* and *C. bracarensis* based on fragment length polymorphism of ITS1 and ITS2 and restriction fragment length polymorphism of ITS and D1/D2 regions in rDNA. *Eur J Clin Microbiol Infect Dis* 30(11)**,** 1409-1416. doi: 10.1007/s10096-011-1235-9.

Sharma, C., Wankhede, S., Muralidhar, S., Prakash, A., Singh, P.K., Kathuria, S., et al. (2013). *Candida nivariensis* as an etiologic agent of vulvovaginal candidiasis in a tertiary care hospital of New Delhi, India. *Diagn Microbiol Infect Dis* 76(1)**,** 46-50. doi: 10.1016/j.diagmicrobio.2013.02.023.

Swoboda-Kopec, E., Sikora, M., Golas, M., Piskorska, K., Gozdowski, D., and Netsvyetayeva, I. (2014). *Candida nivariensis* in comparison to different phenotypes of *Candida glabrata*. *Mycoses* 57(12)**,** 747-753. doi: 10.1111/myc.12264.

Tay, S.T., Lotfalikhani, A., Sabet, N.S., Ponnampalavanar, S., Sulaiman, S., Na, S.L., et al. (2014). Occurrence and characterization of *Candida nivariensis* from a culture collection of *Candida glabrata* clinical isolates in Malaysia. *Mycopathologia* 178(3-4)**,** 307-314. doi: 10.1007/s11046-014-9778-9.

Wahyuningsih, R., SahBandar, I.N., Theelen, B., Hagen, F., Poot, G., Meis, J.F., et al. (2008). *Candida nivariensis* isolated from an Indonesian human immunodeficiency virus-infected patient suffering from oropharyngeal candidiasis. *J Clin Microbiol* 46(1)**,** 388-391. doi: 10.1128/JCM.01660-07.

Warren, T.A., McTaggart, L., Richardson, S.E., and Zhang, S.X. (2010). *Candida bracarensis* bloodstream infection in an immunocompromised patient. *J Clin Microbiol* 48(12)**,** 4677-4679. doi: 10.1128/JCM.01447-10.
