# Supplementary material for: Identification and Antifungal Susceptibility Profiles of Candida nivariensis and Candida bracarensis in a Multi-Center Chinese Collection of Yeasts
Source: Front Microbiol. 2017 Jan 19;8:5. doi: 10.3389/fmicb.2017.00005 (PMC5243801; doi:10.3389/fmicb.2017.00005)
Supplement: Supplementary file 2 [file Table_2.DOCX]

**Table S2 Overview of published reports on antifungal susceptibility profiles of *Candida nivariensis* and *Candida bracarensis***

| **Strain or parameter** | **Region** | **Origin** | **ANF** | **MCF** | **CAS** | **5FC** | **POS** | **VRC** | **ITC** | **FLC** | **AMB** | **ISA** | **Reference** |
| --- | --- | --- | --- | --- | --- | --- | --- | --- | --- | --- | --- | --- | --- |
| ***Candida nivariensis*** | |  |  |  |  |  |  |  |  |  |  |  |  |
| K16661 | Japan | Blood |  | 0.06 |  | 2 |  | 4 | ≥16 | ≥128 | 0.5 |  | ([Fujita et al., 2007](#_ENREF_7)) |
| 13* | UK | Mouth, blood *et al* |  |  | 0.5*^a^* | 0.25 *^a^* | 1 *^a^* | 0.5 *^a^* | 8 *^a^* | 64 *^a^* | 0.5 *^a^* |  | ([Borman et al., 2008](#_ENREF_2)) |
|  |  |  |  |  | 1*^b^* | 0.5 *^b^* | 2 *^b^* | 4 *^b^* | >16 *^b^* | >64 *^b^* | 0.5 *^b^* |  |  |
| CBS 9983 | Iran | NS |  |  | 0.5 | 0.25 | 0.5 | 0.125 | 0.5 | 8 | 1 | 0.031 | ([Wahyuningsih et al., 2008](#_ENREF_14)) |
| CBS 10161 | Indonesia | Oral rinse |  |  | 1 | 0.125 | 0.25 | 0.063 | 0.125 | 2 | 1 | 0.008 | ([Wahyuningsih et al., 2008](#_ENREF_14)) |
| NS | Australia | Pleural fluid | 0.06 | 0.015 | 0.06 |  |  |  |  | 2 | 1 |  | ([Lockhart et al., 2009](#_ENREF_10)) |
| VPCI 32/08 | India | Sputum |  |  |  | 1 |  | 0.03 | 0.25 | 2 | 0.5 |  | ([Chowdhary et al., 2010](#_ENREF_3)) |
|  |  |  |  |  | 0.25*^c^* | 0.094 *^c^* | 0.38 *^c^* | 0.064 *^c^* |  | 2 *^c^* | 0.5 *^c^* |  | ([Chowdhary et al., 2010](#_ENREF_3)) |
| VPCI 1293/08 | India | Blood |  |  |  | 1 |  | 0.03 | 0.25 | 1 | 0.5 |  | ([Chowdhary et al., 2010](#_ENREF_3)) |
|  |  |  |  |  | 0.125 *^c^* | 0.125 *^c^* | 0.38 *^c^* | 0.047 *^c^* |  | 6 *^c^* | 0.5 *^c^* |  | ([Chowdhary et al., 2010](#_ENREF_3)) |
| URCn1 | UK | Urine |  |  |  |  |  |  |  | 8 |  |  | ([Gorton et al., 2013](#_ENREF_8)) |
| URCn2 | UK | Urine |  |  |  |  |  |  |  | 8 |  |  | ([Gorton et al., 2013](#_ENREF_8)) |
| URCn3 | UK | Urine |  |  |  |  |  |  |  | >256 |  |  | ([Gorton et al., 2013](#_ENREF_8)) |
| URCn4 | UK | Urine |  |  |  |  |  |  |  | 4 |  |  | ([Gorton et al., 2013](#_ENREF_8)) |
| URCn5 | UK | Urine |  |  |  |  |  |  |  | 4 |  |  | ([Gorton et al., 2013](#_ENREF_8)) |
| NS | Spain | Blood | 0.015 | 0.015 | 0.125 |  | 0.25 | 0.03 | 0.25 | 4 | 1 |  | ([Lopez-Soria et al., 2013](#_ENREF_11)) |
| 5* | India | Vagina | 0.06-0.125*^d^* | 0.015-0.015 *^d^* | 0.25-0.5 *^d^* | 0.125-2 *^d^* | 0.015-0.25 *^d^* | 0.03-0.5 *^d^* | 0.03-0.5 *^d^* | 0.5-16 *^d^* | 0.03-0.125 *^d^* | 0.015-0.25 *^d^* | ([Sharma et al., 2013](#_ENREF_12)) |
| 7* | China | Vagina |  | | | | | | S/SDD | S/SDD |  |  | ([Li et al., 2014](#_ENREF_9)) |
| UMMC 139 | Malaysia | Blood |  |  | 0.047 *^c^* |  |  | 0.032 *^c^* |  | 0.75 *^c^* | 0.064 *^c^* |  | ([Tay et al., 2014](#_ENREF_13)) |
| UMMC 145 | Malaysia | Vagina |  |  | 0.028 *^c^* |  |  | 0.023 *^c^* |  | 1.5 *^c^* | 0.016 *^c^* |  | ([Tay et al., 2014](#_ENREF_13)) |
| NS | China | Toenails |  |  |  |  | 0.125 | 0.25 | 2 | 4 |  |  | ([Feng et al., 2015](#_ENREF_5)) |
| 89339/INCA | Brazil | Nasal secretion |  |  | 0.03 |  | 2 | 0.25 | 0.25 | ≥64 | 0.25 |  | ([Figueiredo-Carvalho et al., 2016](#_ENREF_6)) |
| ***Candida bracarensis*** | |  |  |  |  |  |  |  |  |  |  |  |  |
| 153M^T^ | Portugal | Vaginal exudate |  |  | 1 | 0.063 | 0.25 | 0.063-0.125 | 0.25 | 4 | 0.5-1 | 0.016 | ([Wahyuningsih et al., 2008](#_ENREF_14)) |
| Cagl-78 | USA | Stool |  |  | 0.25 | 0.12 | 0.5 | 0.06 | 0.25 | 8 | 1 |  | ([Bishop et al., 2008](#_ENREF_1)) |
| Cagl-112 | USA | Abscess |  |  | 0.25 | 0.12 | 0.25 | 0.06 | 0.25 | 4 | 0.094 |  | ([Bishop et al., 2008](#_ENREF_1)) |
| Cagl-121 | USA | Throat |  |  | 0.12 | 0.25 | >8 | 8 | >16 | 256 | 0.19 |  | ([Bishop et al., 2008](#_ENREF_1)) |
| 153M^T^ | Portugal | Vaginal exudate |  |  | 0.25 | 0.03 | 0.5 | 0.12 | 0.5 | 8 | 0.023 |  | ([Bishop et al., 2008](#_ENREF_1)) |
| NRRL Y-27794 | USA | NS |  |  | 0.12 | 0.03 | 0.5 | 0.25 | 0.5 | 16 | 0.032 |  | ([Bishop et al., 2008](#_ENREF_1)) |
| NS | USA | Sputum | 0.06 | 0.015 | 0.03 |  |  |  |  | 16 | 8 |  | ([Lockhart et al., 2009](#_ENREF_10)) |
| NS | USA | Blood | 0.06 | 0.015 | 0.03 |  |  |  |  | 2 | 1 |  | ([Lockhart et al., 2009](#_ENREF_10)) |
| Clinical isolate 1 | Canada | Blood | 0.03 | ≤0.008 | 0.06 | ≤0.06 | 0.5 | 0.12 | 0.25 | 8 | 0.25 |  | ([Warren et al., 2010](#_ENREF_15)) |
| Clinical isolate 2 | Canada | Blood | 0.03 | ≤0.008 | 0.06 | ≤0.06 | 0.5 | 0.12 | 0.25 | 8 | 0.5 |  | ([Warren et al., 2010](#_ENREF_15)) |
| CNM-CL-7030 | Spain | Catheter exudate | 0.03 | 0.03 | 0.25 | 0.5 | 0.12 | 0.03 | 0.06 | 2 | 0.25 |  | ([Cuenca-Estrella et al., 2011](#_ENREF_4)) |
| CNM-CL-7326 | Spain | Pleural fluid | 0.03 | 0.03 | 0.5 | 0.5 | 0.12 | 0.06 | 0.12 | 4 | 0.5 |  | ([Cuenca-Estrella et al., 2011](#_ENREF_4)) |
| CNM-CL-7380 | Spain | Blood | 0.03 | 0.03 | 0.5 | 0.25 | 0.06 | 0.12 | 0.06 | 4 | 0.25 |  | ([Cuenca-Estrella et al., 2011](#_ENREF_4)) |
| NS | China | Vagina |  | | | | | | S*^e^* | S*^e^* |  |  | ([Li et al., 2014](#_ENREF_9)) |

* Number of isolates

*^a^*MIC_50_

*^b^*MIC_90_

*^c^*Antifungal susceptibility tests performed by E-test (bioMérieux, Marcy l’Etoile, France).

*^d^*MIC range

*^e^*Commercial agar diffusion test obtained from Rosco Laboratory (A/S Rosco, Taastrup, Denmark)

Abbreviations: FLC, fluconazole; VRC, voriconazole; ITC, itraconazole; POS, posaconazole; CAS, caspofungin; MCF, micafungin; ANF, anidulafungin; AMB, amphotericin B; 5FC, 5-flucytosine; ISA, isavuconazole; NS, not stated

**Reference**

Bishop, J.A., Chase, N., Magill, S.S., Kurtzman, C.P., Fiandaca, M.J., and Merz, W.G. (2008). *Candida bracarensis* detected among isolates of *Candida glabrata* by peptide nucleic acid fluorescence in situ hybridization: susceptibility data and documentation of presumed infection. *J Clin Microbiol* 46(2)**,** 443-446. doi: 10.1128/JCM.01986-07.

Borman, A.M., Petch, R., Linton, C.J., Palmer, M.D., Bridge, P.D., and Johnson, E.M. (2008). *Candida nivariensis*, an emerging pathogenic fungus with multidrug resistance to antifungal agents. *J Clin Microbiol* 46(3)**,** 933-938. doi: 10.1128/JCM.02116-07.

Chowdhary, A., Randhawa, H.S., Khan, Z.U., Ahmad, S., Juneja, S., Sharma, B., et al. (2010). First isolations in India of *Candida nivariensis*, a globally emerging opportunistic pathogen. *Medical Mycology* 48(2)**,** 416-420. doi: 10.3109/13693780903114231.

Cuenca-Estrella, M., Gomez-Lopez, A., Isla, G., Rodriguez, D., Almirante, B., Pahissa, A., et al. (2011). Prevalence of *Candida bracarensis* and *Candida nivariensis* in a Spanish collection of yeasts: comparison of results from a reference centre and from a population-based surveillance study of candidemia. *Med Mycol* 49(5)**,** 525-529. doi: 10.3109/13693786.2010.546373.

Feng, X., Ling, B., Yang, X., Liao, W., Pan, W., and Yao, Z. (2015). Molecular identification of *Candida* species isolated from onychomycosis in Shanghai, China. *Mycopathologia* 180(5-6)**,** 365-371. doi: 10.1007/s11046-015-9927-9.

Figueiredo-Carvalho, M.H., Ramos Lde, S., Barbedo, L.S., Chaves, A.L., Muramoto, I.A., Santos, A.L., et al. (2016). First description of *Candida nivariensis* in Brazil: antifungal susceptibility profile and potential virulence attributes. *Mem Inst Oswaldo Cruz* 111(1)**,** 51-58. doi: 10.1590/0074-02760150376.

Fujita, S., Senda, Y., Okusi, T., Ota, Y., Takada, H., Yamada, K., et al. (2007). Catheter-related fungemia due to fluconazole-resistant *Candida nivariensis*. *J Clin Microbiol* 45(10)**,** 3459-3461. doi: 10.1128/JCM.00727-07.

Gorton, R.L., Jones, G.L., Kibbler, C.C., and Collier, S. (2013). *Candida nivariensis* isolated from a renal transplant patient with persistent candiduria-molecular identification using ITS PCR and MALDI-TOF. *Med Mycol Case Rep* 2**,** 156-158. doi: 10.1016/j.mmcr.2013.10.001.

Li, J., Shan, Y., Fan, S., and Liu, X. (2014). Prevalence of *Candida nivariensis* and *Candida bracarensis* in vulvovaginal Candidiasis. *Mycopathologia* 178(3-4)**,** 279-283. doi: 10.1007/s11046-014-9800-2.

Lockhart, S.R., Messer, S.A., Gherna, M., Bishop, J.A., Merz, W.G., Pfaller, M.A., et al. (2009). Identification of *Candida nivariensis* and *Candida bracarensis* in a large global collection of *Candida glabrata* isolates: comparison to the literature. *J Clin Microbiol* 47(4)**,** 1216-1217. doi: 10.1128/JCM.02315-08.

Lopez-Soria, L.M., Bereciartua, E., Santamaria, M., Soria, L.M., Hernandez-Almaraz, J.L., Mularoni, A., et al. (2013). First case report of catheter-related fungemia by *Candida nivariensis* in the Iberian Peninsula. *Rev Iberoam Micol* 30(1)**,** 69-71. doi: 10.1016/j.riam.2012.09.001.

Sharma, C., Wankhede, S., Muralidhar, S., Prakash, A., Singh, P.K., Kathuria, S., et al. (2013). *Candida nivariensis* as an etiologic agent of vulvovaginal candidiasis in a tertiary care hospital of New Delhi, India. *Diagn Microbiol Infect Dis* 76(1)**,** 46-50. doi: 10.1016/j.diagmicrobio.2013.02.023.

Tay, S.T., Lotfalikhani, A., Sabet, N.S., Ponnampalavanar, S., Sulaiman, S., Na, S.L., et al. (2014). Occurrence and characterization of *Candida nivariensis* from a culture collection of *Candida glabrata* clinical isolates in Malaysia. *Mycopathologia* 178(3-4)**,** 307-314. doi: 10.1007/s11046-014-9778-9.

Wahyuningsih, R., SahBandar, I.N., Theelen, B., Hagen, F., Poot, G., Meis, J.F., et al. (2008). *Candida nivariensis* isolated from an Indonesian human immunodeficiency virus-infected patient suffering from oropharyngeal candidiasis. *J Clin Microbiol* 46(1)**,** 388-391. doi: 10.1128/JCM.01660-07.

Warren, T.A., McTaggart, L., Richardson, S.E., and Zhang, S.X. (2010). *Candida bracarensis* bloodstream infection in an immunocompromised patient. *J Clin Microbiol* 48(12)**,** 4677-4679. doi: 10.1128/JCM.01447-10.
